# Probing the diversity of chloromethane-degrading bacteria by comparative genomics and isotopic fractionation

**DOI:** 10.3389/fmicb.2014.00523

**Published:** 2014-10-15

**Authors:** Thierry Nadalig, Markus Greule, Françoise Bringel, Frank Keppler, Stéphane Vuilleumier

**Affiliations:** ^1^Université de Strasbourg, Equipe Adaptations et Interactions Microbiennes dans l’Environnement, Unitès Mixtes de Recherche 7156 Centre National de la Recherche Scientifique, Génétique Moléculaire, Génomique, MicrobiologieStrasbourg, France; ^2^Institute of Earth Sciences, Ruprecht Karls University HeidelbergHeidelberg, Germany

**Keywords:** bacteria, chloromethane, comparative genomics, isotope fractionation, diversity

## Abstract

Chloromethane (CH_3_Cl) is produced on earth by a variety of abiotic and biological processes. It is the most important halogenated trace gas in the atmosphere, where it contributes to ozone destruction. Current estimates of the global CH_3_Cl budget are uncertain and suggest that microorganisms might play a more important role in degrading atmospheric CH_3_Cl than previously thought. Its degradation by bacteria has been demonstrated in marine, terrestrial, and phyllospheric environments. Improving our knowledge of these degradation processes and their magnitude is thus highly relevant for a better understanding of the global budget of CH_3_Cl. The *cmu* pathway, for *c*hloro*m*ethane *u*tilisation, is the only microbial pathway for CH_3_Cl degradation elucidated so far, and was characterized in detail in aerobic methylotrophic Alphaproteobacteria. Here, we reveal the potential of using a two-pronged approach involving a combination of comparative genomics and isotopic fractionation during CH_3_Cl degradation to newly address the question of the diversity of chloromethane-degrading bacteria in the environment. Analysis of available bacterial genome sequences reveals that several bacteria not yet known to degrade CH_3_Cl contain part or all of the complement of *cmu* genes required for CH_3_Cl degradation. These organisms, unlike bacteria shown to grow with CH_3_Cl using the *cmu* pathway, are obligate anaerobes. On the other hand, analysis of the complete genome of the chloromethane-degrading bacterium *Leisingera methylohalidivorans* MB2 showed that this bacterium does not contain *cmu* genes. Isotope fractionation experiments with *L. methylohalidivorans* MB2 suggest that the unknown pathway used by this bacterium for growth with CH_3_Cl can be differentiated from the *cmu* pathway. This result opens the prospect that contributions from bacteria with the *cmu* and *Leisingera*-type pathways to the atmospheric CH_3_Cl budget may be teased apart in the future.

## INTRODUCTION

Halocarbons such as chloromethane (CH_3_Cl) and bromomethane are known for their ozone depletion potential (Harper, 2000). CH_3_Cl, the most abundant volatile halocarbon in the atmosphere (∼600 ppt), is responsible for approximately 15% of halogen-dependent ozone destruction in the stratosphere (Harper, 2000; Montzka and Reimann, 2011). The largest sources of CH_3_Cl emissions to the atmosphere include terrestrial vegetation (Hamilton et al., 2003; Yoshida et al., 2004; Keppler et al., 2005) and in particular the phyllosphere (i.e., aboveground parts of vegetation, Saito and Yokouchi, 2008), biomass burning, and the oceans (Montzka and Reimann, 2011). Conversely, the dominant sink for CH_3_Cl is via reaction with hydroxyl radicals in the troposphere and represents 84% of the total, estimated at 4.1 Tg Cl yr^-1^ (Yoshida et al., 2004). However, certain methylotrophic bacteria capable of using CH_3_Cl as their sole source of carbon and energy for growth may also participate in this process, but the magnitude of their contribution remains to be characterized. Chloromethane-degrading bacteria are quite widespread, with representatives affiliated to the genera *Aminobacter*, *Hyphomicrobium, Leisingera, Methylobacterium*, *Roseovarius* (Alpha-Proteobacteria), *Pseudomonas* (Gamma-Proteobacteria*)* and *Acetobacterium* (*Actinobacteria),* isolated from diverse environments such as soils (Doronina et al., 1996; Miller et al., 1997; Coulter et al., 1999; McAnulla et al., 2001), activated sludge (Hartmans et al., 1986; Traunecker et al., 1991; Freedman et al., 2004), freshwaters (McAnulla et al., 2001), and seawater (Schäfer et al., 2005).

The only pathway for CH_3_Cl degradation known so far is corrinoid- and tetrahydrofolate-dependent, and was characterized in detail for the aerobic facultative methylotrophic strain *Methylobacterium extorquens* CM4 (Vannelli et al., 1999). This pathway, termed *cmu* (abbreviation for chloromethane utilization), involves a set of genes that were subsequently detected in several other chloromethane-degrading strains (reviewed in Schäfer et al., 2007; also see Nadalig et al., 2011). The first step of the *cmu* pathway involves the methyltransferase/corrinoid-binding CmuA protein, which transfers the CH_3_Cl methyl group to a corrinoid cofactor, and CmuB, another methyltransferase which catalyzes the transfer of the methyl group from the methylated corrinoid to tetrahydrofolate (H_4_F). Methyl-H_4_F is then oxidized to methylene-H_4_F and further to CO_2_ via formate to conserve energy, or exploited for biomass production. However, other yet to be characterized metabolic pathways may be involved in the degradation of CH_3_Cl in the environment. For example, *Leisingera methylohalidivorans* MB2 grows with methyl halides but was reported not to contain close homologs of *cmu* genes (Schäfer et al., 2007).

Evidence for a given metabolic pathway may be obtained through the use of stable isotope techniques, and this has been used to distinguish different sources and sinks for CH_3_Cl (Harper et al., 2001, 2003; Czapiewski et al., 2002; Keppler et al., 2004, 2005; Saito and Yokouchi, 2008; Greule et al., 2012; Redeker and Kalin, 2012). Degradation of CH_3_Cl by cell suspensions of strains with the *cmu* pathway is also associated with specific carbon fractionation (Miller et al., 2001) but also with hydrogen isotope fractionation (Nadalig et al., 2013). Thus, isotopic approaches combined with genomic approaches may prove decisive in constraining the bacterial contribution to the global CH_3_Cl budget.

In the present study, we review available bacterial genome sequences for the presence of *cmu* genes, thereby uncovering several bacteria that have not been described to degrade CH_3_Cl. In parallel and as a proof of concept for the potential of isotope methods to characterize yet unknown pathways for CH_3_Cl degradation, we determined hydrogen and carbon isotopic fractionation patterns of CH_3_Cl during growth of the chloromethane-degrading strain *L. methylohalidivorans* MB2 lacking *cmu* genes, as compared to that observed for *cmu* pathway strains *M. extorquens* CM4 and *Hyphomicrobium* sp. MC1.

## MATERIALS AND METHODS

### BIOINFORMATIC ANALYSIS

Comparative genome analysis was performed with the software tools available on the Microscope platform at Genoscope (Vallenet et al., 2009), using the assembled sequences of *M. extorquens* CM4 (GenBank accession numbers CP001298, CP001299, CP001300), *Hyphomicrobium* sp. MC1 (FQ859181), *Desulfomonile tiedjei* (CP003360, CP003361), *Thermosediminibacter oceani* (CP002131), *Thermincola potens* (CP002028), and *L. methylohalidivorans* MB2 (CP006773, CP006774, CP006775), and the draft sequences for *Desulfotomaculum alcoholivorax* (GenBank AUMW00000000; 66 contigs), *Desulfurispora thermophila* (GenBank AQWN00000000; 19 contigs; **Table 1**).

**Table 1:** Characteristics of the bacterial strains discussed in this study.

Strains	*Methylobacterium extorquens* CM4	*Hyphomicrobium* sp. MC1	*Desulfotomaculum alcoholivorax* DSM 16058	*Desulfurispora thermophila* DSM 16022	*Desulfomonile tiedjei* DSM 6799	*Thermincola potens* JR	*Thermosedimi-nibacter oceani* DSM 16646	*Leisingera methylohalidivorans* MB2 DSM 14336
Affiliation	Alphaproteobacteria	Alphaproteobacteria	Deltaproteobacteria	Deltaproteobacteria	Deltaproteobacteria	Clostridia	Clostridia	Alphaproteobacteria
Origin	Industrial soil	Sewage sludge	Fluidized-bed reactor	Fluidized-bed reactor	Sewage sludge	Anodic biofilm	Deep-sea sediment	Sea water
Genome size (MB)	6.18	4.75	3.47	2.82	6.53	3.16	2.28	4.65
GC (%)	68	59	47	55	50	46	47	62
Total CDS	6035	4955	3588	2815	5664	3343	2460	4608
Plasmids	2	0	Unknown	Unknown	1	0	0	2
Sequence status	Assembled, finished	Assembled, finished	66 contigs Permanent draft	19 contigs Permanent draft	Assembled, finished	Assembled, finished	Assembled, finished	Assembled, finished
Genbank accession number	CP001298 CP001299 CP001300	FQ859181	AUMW00000000	AQWN00000000	CP003360 CP003361	CP002028	CP002131	CP006773 CP006774 CP006775
Reference	Marx et al. (2012)	Vuilleumier et al. (2011)	Kaksonen et al. (2008)	Kaksonen et al. (2007)	DeWeerd et al. (1990)	Byrne-Bailey et al. (2010)	Pitluck et al. (2010)	Buddruhs et al. (2013)

### BACTERIAL STRAINS AND GROWTH CONDITIONS

Strains *M. extorquens* CM4 and *Hyphomicrobium* sp. MC1 were laboratory stocks and cultivated in a mineral medium for methylotrophic bacteria (M3; Roselli et al., 2013) containing (L^-1^ of distilled water) KH_2_PO_4_ (6.8 g), (NH_4_)_2_SO_4_ (0.2 g), NaOH (5 M) (5.85 mL), yielding a final pH of 7.2. After autoclaving, 1 mL L^-1^ medium each of calcium nitrate solution (25 g L^-1^) and of trace elements solution containing (mg L^-1^) FeSO_4_ 7H_2_O (100), MnSO_4_ H_2_O (100), ZnSO_4_ (29.5), Co(NO_3_)_2_ 6H_2_O (25), CuCl_2_ H_2_O (25), Na_2_MoO_4_ 2H_2_O (25), NH_4_VO_3_ (14.4), NiSO_4_ 6H_2_O (10), H_3_BO_3_ (10), and 0.5 mL L^-1^of H_2_SO_4_ (95%) were added. Strain *L. methylohalidivorans* MB2 (DSM 14336) was obtained from DSMZ (Braunschweig, Germany) and cultivated in a mineral medium (MAMS) containing (L^-1^ of distilled water) NaCl (16 g), (NH_4_)_2_SO_4_ (1 g), MgSO_4_ 7H_2_O (1 g), CaCl_2_ 2H_2_O (0.2 g), KH_2_PO_4_ (0.36 g), and K_2_HPO_4_ (2.34 g) as described (Schaefer et al., 2002). After autoclaving, 1 mL L^-1^ medium of trace elements solution was added. Strains CM4, MC1, and MB2 were grown with CH_3_Cl gas [10 mL (Fluka), effectively yielding approximately 10 mM final concentration], in 300 mL Erlenmeyer vials fitted with sealed mininert valve caps (Sigma) and containing 50 mL of medium. Cultures were incubated at 30°C on a rotary shaker (100 rpm). Abiotic controls (no bacteria added) were prepared and incubated in the same way. Growth was followed by absorbance measurement at 600 nm.

The headspace of cultures was sampled regularly (0.1 mL) for determination of CH_3_Cl concentration by gas chromatography, and 1 mL headspace samples were also taken at each point and conserved in 12 ml Exetainers® (Labco Limited, Lampeter, UK) for subsequent isotopic measurements. Concentration of chloride was measured in supernatants of cultures using the spectrophotometric method of Jörg and Bertau (2004), except for *L. methylohalidivorans* MB2 because of the high chloride content of MAMS medium.

### ANALYSIS OF CONCENTRATIONS AND STABLE ISOTOPE VALUES OF CHLOROMETHANE

Concentration and stable carbon and hydrogen isotope values for CH_3_Cl were performed by gas chromatography coupled with flame ionization detector (GC-FID) and isotope ratio mass spectrometry (IRMS), respectively, as described previously (Nadalig et al., 2013), except that helium flow entering the gas chromatograph in isotopic analysis was increased to 1.8 ml min^-1^.

The conventional “delta” notation, which expresses the isotopic composition of a material relative to that of a standard on a per mil (‰) deviation basis, was used. Values of δ^2^H (‰) are relative to that for V-SMOW (Vienna Standard Mean Ocean Water), and values of δ^13^C (‰) are relative to that for V-PDB (Vienna Pee Dee Belemnite). Carbon and hydrogen isotope fractionations associated with CH_3_Cl degradation by *L. methylohalidivorans* MB2, *M. extorquens* CM4 and *H.* sp. MC1 were determined from the slopes (*b_C_ and b_H_*) of the linear regression of isotope variation (^13^C and δ^2^H) of CH_3_Cl on the logarithm of the remaining CH_3_Cl concentration (ln f):

$$b_{C}=\delta^{13}C/\ln\;f\,\;\;and\,\;\;\;b_{H}=\delta^{2}H/\ln\;f.$$

Fractionation factors α_C_ and α_H_ were calculated as α = 1,000/(*b*+1,000), and also expressed as isotope enrichment factors (ε_C_ and ε_H_), calculated as ε = (α-1)10^3^. Errors represent 95% confidence intervals calculated on the least-squares regression.

## RESULTS

**FIGURE 1:**
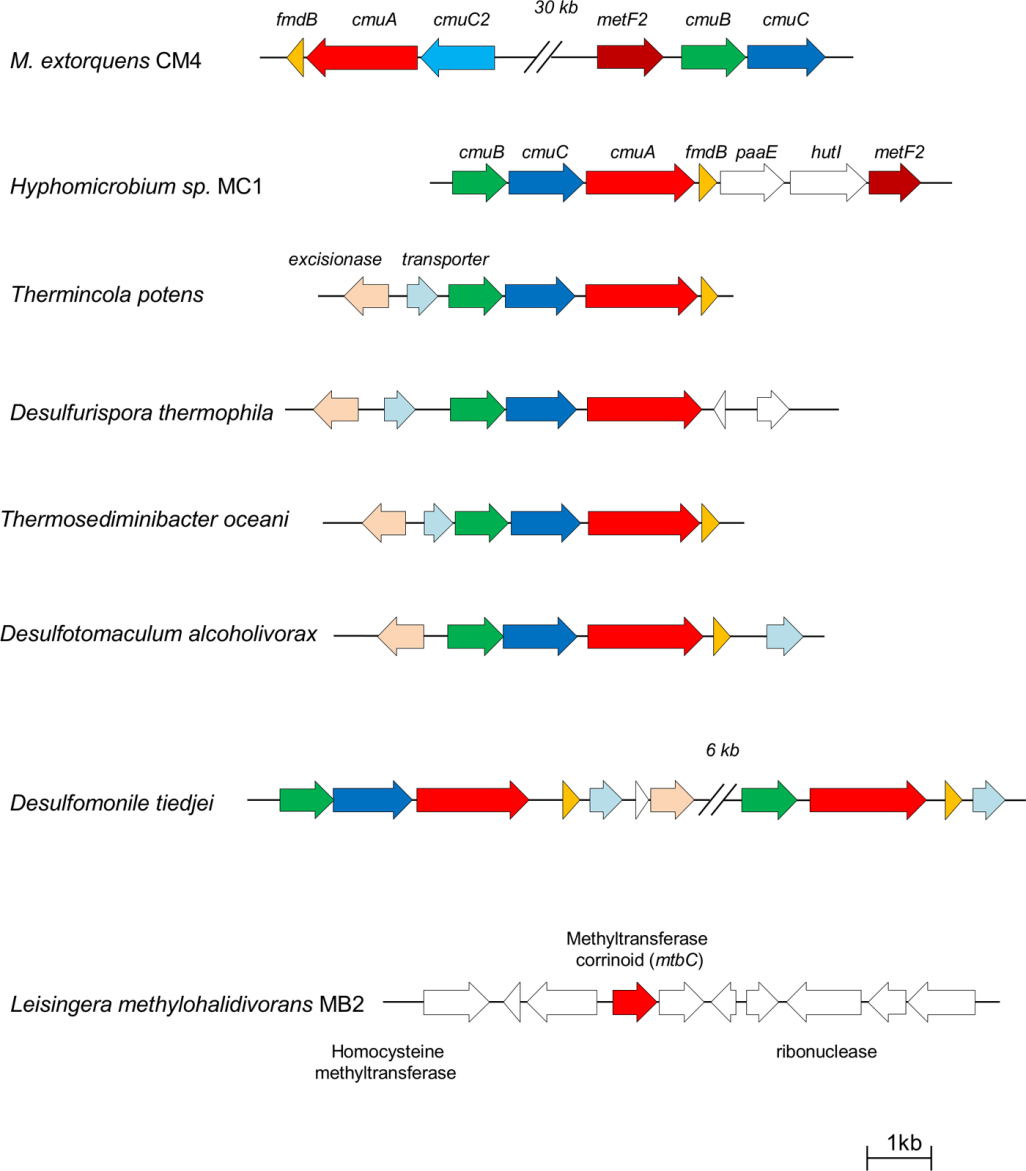
**Comparison of *cmu* gene organization in sequenced genomes of chloromethane-degrading bacteria.** Arrows represent protein-coding genes, and homologous genes are shown in the same color. Gene clusters are drawn to scale.

Several chloromethane-degrading bacteria with the *cmu* pathway have been characterized (Schäfer et al., 2007), and a complete and assembled genome sequence is available for two of them, i.e., *M. extorquens* CM4 (Marx et al., 2012) and *Hyphomicrobium* sp. MC1 (Vuilleumier et al., 2011). Two types of organization of *cmu* genes were identified (Nadalig et al., 2011). The usual gene organization involves a putative *cmuBCA* operon and was found in all experimentally characterized chloromethane-degrading bacteria with the *cmu* pathway except the reference chloromethane-degrading strain *M. extorquens* CM4, which harbors *cmu* genes in two clusters (**Figure 1**). The chloromethane-degrading strain *L. methylohalidivorans* MB2, in contrast, was known to lack *cmu* genes (Schäfer et al., 2007), so the recent report of its assembled genome sequence (Buddruhs et al., 2013) was of particular interest.

### COMPARATIVE GENOMICS

An exhaustive survey of the presence of *cmu* genes in available sequenced bacterial genomes was carried out, yielding several novel insights (**Table 2**). First, all strains with *cmu* homologs contained all three genes *cmuA*, *cmuB,* and *cmuC* essential for growth with CH_3_Cl using the *cmu* pathway. Second, these three genes were detected as a *cmuBCA* gene cluster (**Figure 1**) in the genomes of five bacterial strains that had not been reported to possess *cmu* genes or CH_3_Cl degradation activity. Strikingly and in contrast to all strains growing with CH_3_Cl using the *cmu* pathway described so far, all these strains are anaerobes. Three of them are Gram-negative bacteria from the class Deltaproteobacteria, i.e., *Desulfotomaculum alcoholivorax* (Kaksonen et al., 2008), *Desulfurispora thermophila* (Kaksonen et al., 2007) and *Desulfomonile tiedjei* (DeWeerd et al., 1990), and two belong to the class Clostridia, i.e., the Gram-positive *Thermincola potens* (Byrne-Bailey et al., 2010) and the Gram-negative *Thermosediminibacter oceani* (Pitluck et al., 2010). Notably, *Desulfomonile tiedjei* has a second *cmu* cluster containing only *cmuB* and *cmuA* (**Figure 1**) 6 kb away from a *cmuBCA* gene cluster. Levels of identity with homologs of the CM4 strain at the protein level are high, and range between 64 and 84%, 60 and 64%, and 34 and 39% for *cmuA*, *cmuB* and *cmuC* gene products, respectively (**Table 2**). Pairwise identity comparisons of the proteins encoded by *cmu* genes show that homologs of strains *Desulfotomaculum alcoholivorax*, *Desulfurispora thermophila*, *Thermincola potens* and *Thermosediminibacter oceani* are most related to each other, with identities at the protein level between 84–93%, 82–92%, and 66–87% for *cmuA*, *cmuB,* and *cmuC* gene products, respectively, and that CM4 homologs represent outliers for all three genes. It is interesting to note that the gene products of two copies of c*muA* and *cmuB* of *Desulfomonile tiedjei* (78 and 77% protein identity, respectively) are not each others’ closest homologs. In addition, no evidence for substantial identity at the DNA level was detected between the two *cmu* gene clusters of this strain (data not shown). Further, CmuA encoded by the *cmuBCA* cluster of *Desulfomonile tiedjei* is closer to homologs from other strains (>80% identity at the protein level) than that encoded by the partial *cmu* cluster *cmuBA* (<75% identity).

**Table 2:** Key CDS related to the *cmu* pathway in investigated genomes^a^.

Protein	*Methylobacterium extorquens* CM4^b^	*Hyphomicrobium* sp. MC1	*Desulfotomaculum alcoholivorax* DSM 16058	*Desulfurispora thermophila* DSM 16022	*Desulfomonile tiedjei* DSM 6799	*Thermincola potens* JR	*Thermosediminibacter oceani* DSM 16646	*Leisingera methylohalidivorans* MB2 DSM 14336
CmuA	Mchl_5697	HYPMCv2_2273 (84%)	DESALv160093 (69%)	AQWNv1_40006 (67%)	Desti_5447 (67%) Desti_5437 (64%)	TherJR_0143 (68%)	Toce_1533 (68%)	Leime_2531^c^ (32%)
CmuB	Mchl_5727	HYPMCv2_2275 (64%)	DESALv160091 (62%)	AQWNv1_40005 (62%)	Desti_5449 (60%) Desti_5438 (60%)	TherJR_0145 (61%)	Toce_1535 (62%)	n.d.^d^
CmuC CmuC2	Mchl_5728 Mchl_5698	HYPMCv2_2274 (39/35%)^e^	DESALv160092 (36/38%)	AQWNv1_40004 (35/34%)	Desti_5448 (37/36%)	TherJR_0144 (37/37%)	Toce_1534 (35/35%)	n.d.
FmdB	Mchl_5696	HYPMCv2_2271 (45%)	DESALv160094 (38%)	n.d.	Desti_5446 (42%) Desti_5436 (43%)	TherJR_0142 (42%)	Toce_1532 (37%)	n.d.
FolD	Mchl_5700	HYPMCv2_2266 (47%)	DESALv110219 (40%)	AQWNv1_30228 (42%)	Desti_2379 (46%)	TherJR_1709 (41%) TherJR_1706 (39%)	Toce_0805 (40%)	Leime_3180 (52%) Leime_4077 (52%)
HutI	Mchl_5694	HYPMCv2_2269 (53%)	DESALv150151 (33%)	AQWNv1_70146 (34%)	n.d.	n.d.	Toce_1473 (33%)	Leime_0109 (39%)
MetF2	Mchl_5726	HYPMCv2_2268 (31%)	n.d.	n.d.	n.d.	n.d.	n.d.	Leime_2796 (35%)
MetF	Mchl_1881	HYPMCv2_2119 (67%)	n.d.	n.d.	n.d.	n.d.	n.d.	Leime_1763 (46%)
PurU	Mchl_5699	HYPMCv2_2267 (62%)	DESALv110045 (34%)	AQWNv1_20249 (32%)	n.d.	TherJR_0829 (33%)	Toce_1499 (33%)	Leime_2536 (36%)
Serine pathway (10 reactions)	Complete	Complete	4	4	5	3	5	9
Ethylmalonyl-CoA pathway (14 reactions)	Complete	Complete	10	9	10	2	6	Complete
H_4_F pathway (3 reactions)	Complete	Complete	Complete	Complete	Complete	Complete	Complete	Complete
H_4_MPT pathway (7 reactions)	Complete	Complete	0	0	0	0	0	0

*^a^Sequence identity (>30%) at the protein level to M. extorquens CM4 CDS in brackets. All CDS homologs of CM4 genes are chromosomally encoded except hutI in L. methylohalidivorans MB2 which is plasmid-encoded.*

*^b^All CM4 CDS are plasmid-encoded except metF.*

*^c^Over the full length (211 aa) of the homolog.*

*^d^n.d., not detected.*

*^e^Sequence identity at the protein level to CmuC/CmuC2 of M. extorquens CM4 in brackets.*

Analysis of the *L. methylohalidivorans* MB2 genome (Buddruhs et al., 2013) confirmed the original report of Schäfer et al. (2007) that this CH_3_Cl strain did not contain *bona fide cmu* genes. As mentioned in the genome report, the closest homolog to *cmuA* is a gene coding a short (232 residues) corrinoid methyltransferase protein (MtbC) with only 32% identity to the *C*-terminal domain of CmuA (**Table 2**). However, no full-length homologs to *cmuB* and *cmuC* were detected in the genome sequence (**Table 2**). Taken together, these data confirm that the metabolic pathway used by *L. methylohalidivorans* MB2 to grow with CH_3_Cl is different to that of other known chloromethane-degrading strains with the *cmu* pathway.

The presence of downstream genes in the *cmu* pathway in strains containing *cmuABC* genes was also evaluated (**Table 2**). Genes potentially involved in the tetrahydrofolate (H_4_F) dependent pathway for oxidation of methyl-H_4_F to formate via methylene-H_4_F are present in all genomes (**Table 2**), but close homologs of *metF* encoding methylene-H_4_F reductase were not detected except in strain MC1. Notably, only Alphaproteobacterial strains CM4, MC1, and *L. methylohalidivorans* MB2 possess the genes involved in the serine and ethylmalonyl-CoA pathways involved in growth of strains CM4 and MC1 with C1 compounds. Moreover, the tetrahydromethanopterin (H_4_MPT) pathway crucial for growth of *Methylobacterium* with methanol (Marx et al., 2005), but thought to be dispensable for growth with CH_3_Cl (Studer et al., 2002), is only present in *M. extorquens* CM4 and *Hyphomicrobium* sp. MC1 which also grow with methanol, but absent in *L. methylohalidivorans* MB2, which is unable to grow with methanol, as well as in all other strains containing *cmu* genes investigated here. Finally, a search for genes common to chloromethane-degrading strains (including or excluding *L. methylohalidivorans* MB2) failed to reveal genes other than essential housekeeping genes (data not shown). This suggests that identification of the genes involved in CH_3_Cl degradation or in adaptation to CH_3_Cl metabolism is not possible by comparative genomics analysis alone.

### GROWTH OF STRAINS WITH CHLOROMETHANE AS SOLE CARBON AND ENERGY SOURCE

**FIGURE 2:**
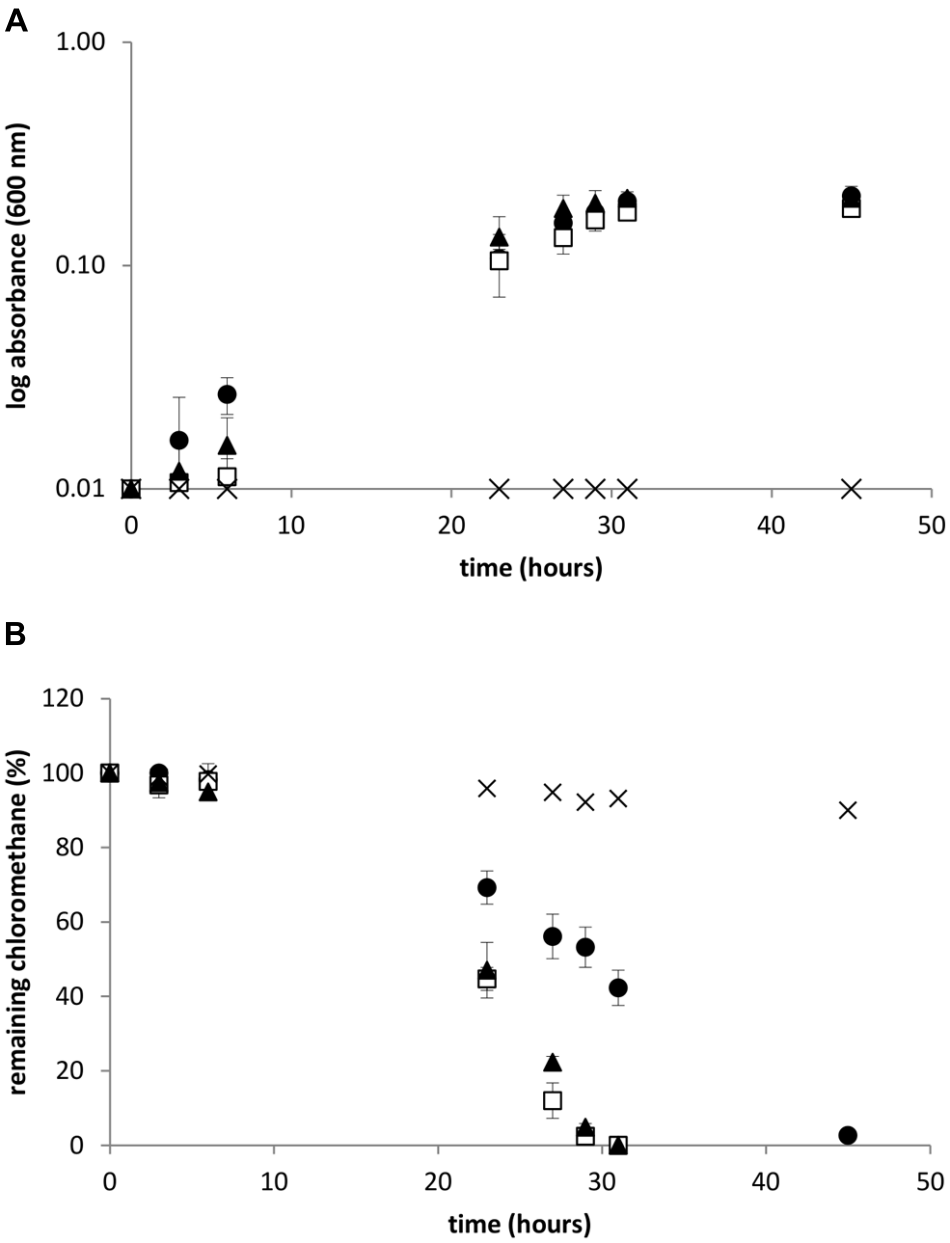
**Growth and chloromethane degradation during bacterial cultivation.** Absorbance at 600 nm **(A)** and consumption of chloromethane (B). *Leisingera methylohalidivorans* MB2 (•), *M. extorquens* CM4 (□) *Hyphomicrobium* sp. MC1 (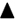) and abiotic control (×). Error bars indicate the standard deviation of the mean of three biological replicates. Chloride concentration at the end of cultivation were 9.2 ± 0.2 and 9.2 ± 0.3 mol L^-1^ for strains CM4 and MC1, respectively, but could not be measured in the high-salt medium used for growth of strains MB2.

*Methylobacterium extorquens* CM4, *Hyphomicrobium* sp. MC1, and *L. methylohalidivorans* MB2 were cultivated with 10 mM CH_3_Cl as sole carbon and energy source in the recommended medium allowing fastest growth, i.e., minimal mineral medium for strains CM4 and MC1, and high-salt mineral medium for strain MB2 (**Figure 2A**). Chloromethane consumption during growth was measured in the gaseous phase by gas chromatography (**Figure 2B**). In cultures of *M. extorquens* CM4 and *H.* sp. MC1, CH_3_Cl was completely degraded after 30 h under the chosen growth conditions. In contrast, consumption of CH_3_Cl by the *L. methylohalidivorans* MB2 culture required a longer time (∼45 h) to proceed to completion, although its growth behavior was similar to that of the other two strains.

### CARBON AND HYDROGEN ISOTOPE FRACTIONATION OF CHLOROMETHANE DURING GROWTH

**FIGURE 3:**
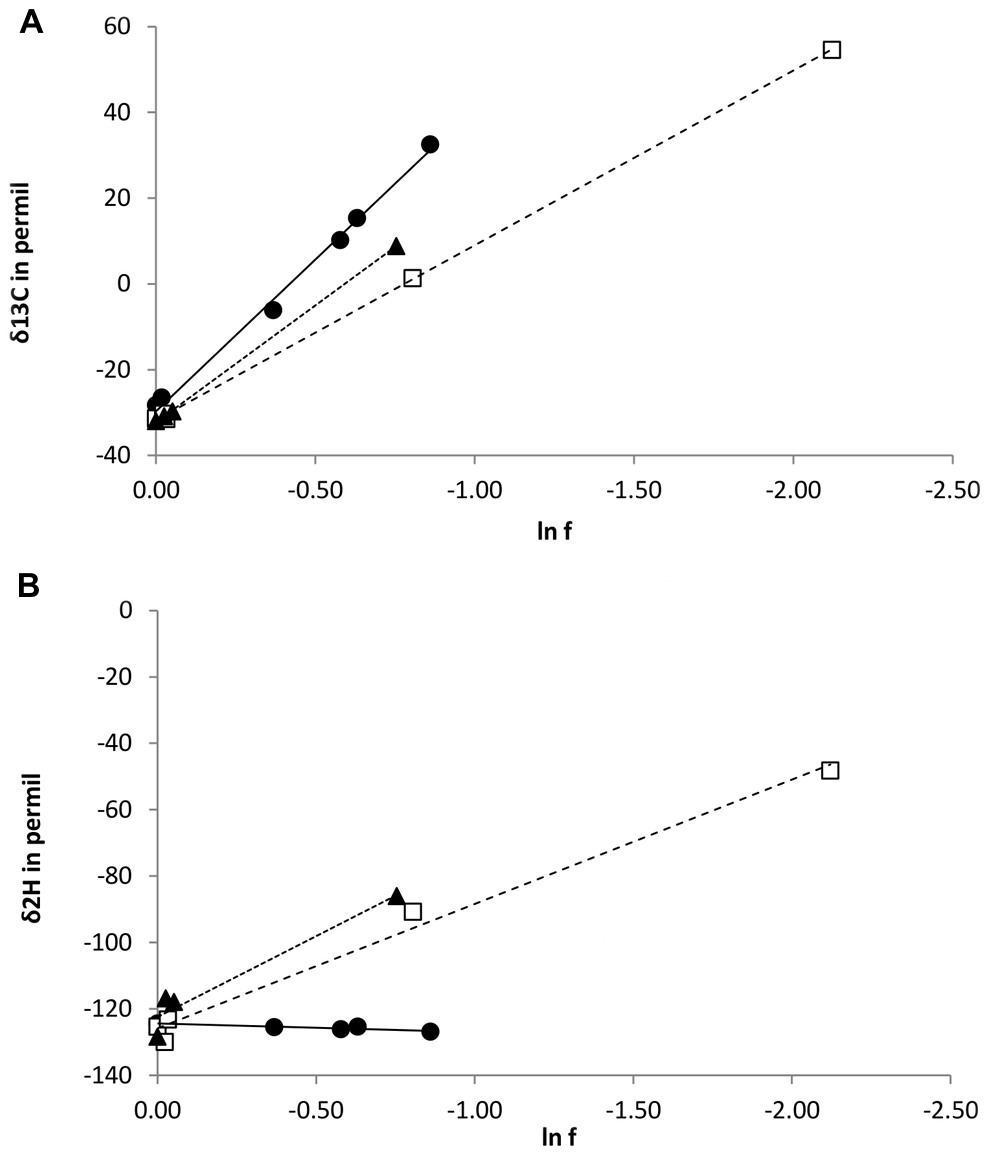
**Isotope variations of chloromethane during bacterial cultivation with chloromethane. (A)** δ^13^C and **(B)** δ^2^H in relation to the fraction of remaining chloromethane (f). Lines represent best-fit linear regressions. *L. methylohalidivorans* MB2 (•), *M. extorquens* CM4 (□), and *Hyphomicrobium* sp. MC1 (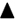).

During degradation of CH_3_Cl, δ^13^C values of residual chloromethane increased from approximately -32‰ (initial value) to 55, 9, and 33‰ for strains CM4, MC1, and MB2 respectively (**Figure 3A**). No carbon or hydrogen fractionation was observed in abiotic controls with media M3 and MAMS (data not shown). Derived values of isotope fractionation factor (α_C_) and of the corresponding enrichment factor were very similar for *cmu* pathway strains CM4 and MC1, and substantially larger for *L. methylohalidivorans* MB2 (**Table 3**).

**Table 3:** Isotopic enrichment (ε) and fractionation (α) factors for carbon and hydrogen during growth with chloromethane.

	ε_c_(‰)	*R*^2a^	α_C_	ε_H_(‰)	*R*^2a^	α_H_
Methylobacterium extorquens CM4	42	0.9997	1.042	39	0.9886	1.039
Hyphomicrobium sp. MC1	54	0.9999	1.054	51	0.9418	1.051
Leisingera methylohalidivorans MB2	76	0.9951	1.076	0	0.8230	1.000

*^a^Quality of fit to linear least-squares regression.*

However, trends were markedly different for the three strains when considering the enrichment of ^2^H in residual CH_3_Cl during cultivation. For strains CM4 and MC1, δ^2^H values increased from approximately -124 at the start of the experiment to 16‰)and -12‰ for strains CM4 and MC1, respectively (**Figure 3B**). In marked contrast, however, no substantial change of δ^2^H was observed during degradation of CH_3_Cl by *L. methylohalidivorans* MB2 (**Figure 3B**). This resulted in large differences of hydrogen stable isotope fractionation factor (α_H_) and of the corresponding enrichment factor between strains CM4 and MC1 containing the *cmu* pathway for CH_3_Cl degradation on the one hand, and strain MB2 lacking the corresponding genes on the other hand (**Table 3**).

## DISCUSSION

The presence of *cmu* genes in recently completed genome sequences was somewhat expected, but their detection in exclusively anaerobic bacteria came as a surprise considering that they had so far only been found in aerobic chloromethane-degrading bacteria. Anaerobic chloromethane-degrading bacteria reported so far use a different, although in one case at least also corrinoid-dependent, pathway (Traunecker et al., 1991; Messmer et al., 1993; Freedman et al., 2004). Worthy of note, CH_3_Cl dehalogenation by the *cmu* pathway does not require aerobic conditions and is actually sensitive to oxygen (Studer et al., 2001). It is thus possible that anaerobic bacteria with *cmu* genes identified here (**Table 2**) are actually able to use CH_3_Cl as a carbon and energy source, although this remains to be tested experimentally.

The conserved *cmuBCA* gene organization (**Figure 1**) in CH_3_Cl-degrading bacteria (Nadalig et al., 2011), and the high level of identity between the protein sequences encoded by *cmu* genes of *Thermincola potens*, *Desulfurispora thermophila*, *Thermosediminibacter oceani*, *Desulfotomaculum alcoholivorax,* and *Desulfomonile tiejdei* (>81, >74, and >58% for *cmuA*, *cmuB,* and *cmuC,* respectively), suggests a common evolutionary origin for these genes and their dissemination in the environment by horizontal gene transfer. The presence of an excisionase in the immediate environment of *cmu* genes in these five strains (**Figure 1**) further supports acquisition of *cmu* genes by horizontal transfer in these strains, as does the presence of two *cmu* gene clusters in *Desulfomonile tiedjei* whose sequences are not closer related to each other than to those of other chloromethane-degrading strains. To our knowledge, however, potential sources of CH_3_Cl that would support dissemination of *cmu* genes in anaerobic environments have not yet been reported.

Incidentally, our analysis also confirms the particular status in the *cmu* pathway of *cmuC*, a gene shown to be essential for growth of strain CM4 with CH_3_Cl (Studer et al., 2002; Roselli et al., 2013) but whose function remains elusive. Indeed, sequence conservation among the proteins encoded by genes *cmuA*, *cmuB,* and *cmuC* are lowest for the CmuC gene product (**Table 2**). Moreover, the probable loss of a *cmuC* homolog in one of the two *cmu* gene clusters of *Desulfomonile tiedjei* strain CM4 (**Figure 1**) also hints at its possibly lesser role in CH_3_Cl metabolism.

As to the chloromethane-degrading strain *L. methylohalidivorans* MB2, analysis of its genome (Buddruhs et al., 2013) confirms the initial report (Schäfer et al., 2007) that it lacks the *cmu* pathway. The best partial hit to CmuA (32% amino acid identity) is a 211-residue corrinoid protein, and *cmuB* or *cmuC* homologs were not detected in the *L. methylohalidivorans* MB2 genome (**Table 2**). However, downstream genes of the H_4_F-dependent *cmu* pathway (*metF*, *folD*, *purU*) were all found, so an H_4_F-dependent metabolic pathway for growth of *L. methylohalidivorans* MB2 with CH_3_Cl remains a possibility.

In our experiments, we showed that *L. methylohalidivorans* MB2, previously grown with CH_3_Cl (0.37 mM; Schaefer et al., 2002), is capable of using this one-carbon compound as sole carbon and energy source at an initial concentration of 10 mM. A direct comparison of its growth behavior with that of strains CM4 and MC1 is prevented by the fact that the latter two strains do not grow in high-salt mineral medium, whereas *L. methylohalidivorans* MB2 does not grow in the standard low-salt mineral medium used for strains CM4 and MC1. Incidentally, this suggests that salt adaptation may be unrelated to adaptation to intracellular chloride production during dehalogenation, as observed recently for bacteria growing with dichloromethane (Michener et al., 2014).

The differences in CH_3_Cl metabolism of *L. methylohalidivorans* MB2 suggested by comparative genomics were experimentally supported by isotope analysis (**Figure 3**; **Table 3**). For *L. methylohalidivorans* MB2, isotopic enrichment factor for carbon during growth was substantially larger than for CM4 and MC1, indicating a larger primary isotope effect and providing further evidence for operation of another pathway for utilization of CH_3_Cl in this strain. In contrast, a previous study on carbon isotopic fractionation of CH_3_Cl by cell suspensions of three bacterial strains, including *L. methylohalidivorans* MB2, gave similar isotopic enrichment values (ranging between 42 and 47‰; Miller et al., 2001). In particular, the value obtained for *Aminobacter ciceronei* strain IMB1, the only strain so far shown to possess *cmuA* but not *cmuB* (Woodall et al., 2001), was similar to those of strains CM4 and MC1 (Miller et al., 2001). This suggests that the corrinoid dehalogenase protein CmuA drives carbon isotopic fractionation in chloromethane-degrading strains with the *cmu* pathway. Moreover and unlike for carbon, a larger isotope effect than in previous resting cell experiments (Nadalig et al., 2013) was observed for hydrogen during growth in strains CM4 and MC1. However, the most striking finding of the present study was the lack of substantial hydrogen isotope enrichment upon CH_3_Cl degradation by *L. methylohalidivorans* MB2. This suggests that unlike CmuAB chloromethane dehalogenase, the unknown dehalogenase of this strain does not cause hydrogen fractionation during degradation of the chloromethane methyl group. Nevertheless and as a common denominator to all three chloromethane-degrading strains investigated here (**Table 3**), carbon isotope fractionation (the primary isotope effect in cleavage of the carbon-halogen bond) was more pronounced than hydrogen isotope fractionation (a secondary isotope effect in CH_3_Cl dehalogenation), as expected (Elsner et al., 2005).

**FIGURE 4:**
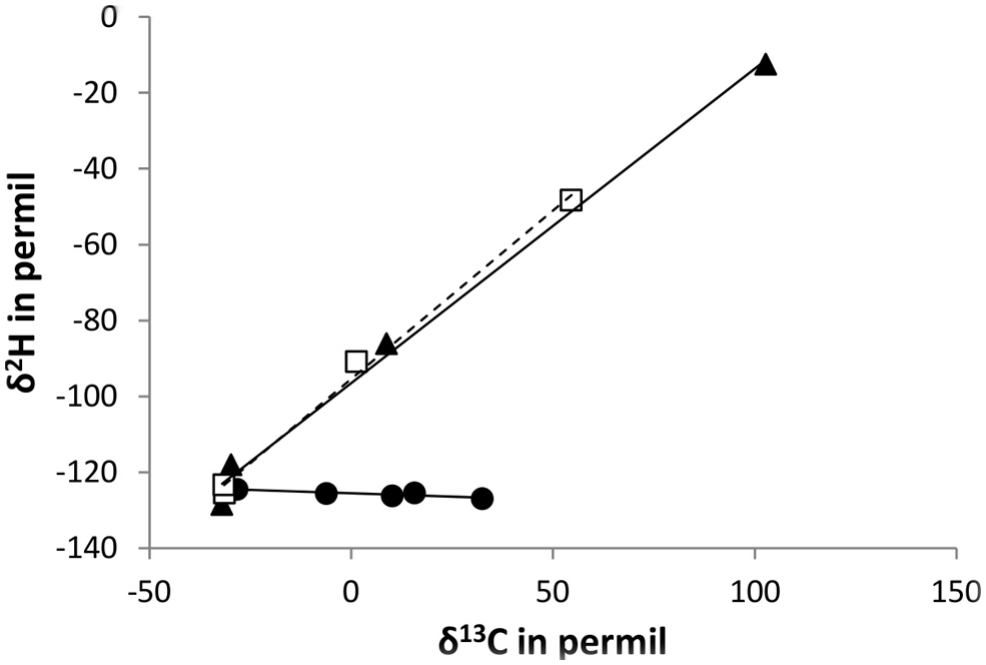
**Changes in carbon and hydrogen isotope ratios for degradation of chloromethane by *L. methylohalidivorans* MB2 (•), *M. extorquens* CM4 (□), and *Hyphomicrobium* sp. MC1 (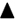) at different time points during growth (increase in time from left to right of the graph).** Lines (slope = -0.03 (*R*^2^: 0.8014) for strain MB2, slope = 0.89 (*R*^2^: 0.9954) for strain CM4, and slope = 0.83 (*R*^2^: 0.9939) for strain MC1, respectively) represent best fit regressions.

The observed differences in isotopic fractionation of CH_3_Cl carbon and hydrogen between the three strains CM4, MC1, and MB2 are best visualized in **Figure 4**, which shows the trends in enrichment of the heavier isotope of carbon and hydrogen for the different strains at different time points during growth. As proposed by Elsner et al. (2005), the slopes in these graphs constitute a clear indication that *L. methylohalidivorans* MB2 uses a different pathway for growth with CH_3_Cl than strains CM4 and MC1, which utilize the same pathway.

Measurements of isotopic fractionation for a given environmental compartment will include the overall contribution of the metabolic diversity of chloromethane-degrading bacteria and their relative occurrence in that environment. It is tempting to speculate that chloromethane degradation in the soil environment, for which an isotopic fractionation of 49‰ similar to that found here for strains CM4 and MC1 was obtained in a previous study (Miller et al., 2004), is predominantly performed by bacteria with the *cmu* pathway. Our results on microbially driven hydrogen and carbon isotope fractionation suggest that using in a two-dimensional isotope scheme might help to confirm this hypothesis. Thus, a combination of genomic studies with physiological and isotopic characterisation of chloromethane-degrading bacterial strains, as performed here, will remain a major objective for the near future in order to constrain the bacterial sink strength of the atmospheric budget of CH_3_Cl.

## Conflict of Interest Statement

The authors declare that the research was conducted in the absence of any commercial or financial relationships that could be construed as a potential conflict of interest.
